# Fast Calibration Methods for Resistive Sensor Readout Based on Direct Interface Circuits

**DOI:** 10.3390/s19183871

**Published:** 2019-09-07

**Authors:** José A. Hidalgo-López, Jesús A. Botín-Córdoba, José A. Sánchez-Durán, Óscar Oballe-Peinado

**Affiliations:** 1 Departamento de Electrónica, Universidad de Málaga, Andalucía Tech, Campus de Teatinos, 29071 Málaga, Spain; 2 Instituto de Investigación Biomédica de Málaga (IBIMA), 29010 Málaga, Spain

**Keywords:** direct interface circuits, calibration methods, error analysis, resistive sensor, interface sensor, time-based measurement

## Abstract

A simple method to measure the resistance of a sensor and convert it into digital information in a programmable digital device is by using a direct interface circuit. This type of circuit deduces the value of the resistor based on the discharge time through it for a capacitor of a known value. Moreover, the discharge times of this capacitor should be measured through one or two resistors with known values in order to ensure that the estimate is not dependent on certain parameters that change with time, temperature, or aging. This can slow down the conversion speed, especially for high resistance values. To overcome this problem, we propose a modified process in which part of the discharge, which was previously performed through the resistive sensor only, is only conducted with the smallest calibration resistor. Two variants of this operation method, which differ in the reduction of the total time necessary for evaluation and in the uncertainty of the measurements, are presented. Experiments carried out with a field programmable gate array (FPGA); using these methodologies achieved reductions in the resistance conversion time of up to 55%. These reductions may imply an increase in the uncertainty of the measurements; however, the tests carried out show that with a suitable choice of parameters, the increases in uncertainty, and therefore errors, may be negligible compared to the direct interface circuits described in the literature.

## 1. Introduction

More and more digital systems are receiving information from the outside world through sensors, making it important to design simple methods that transfer the analogue information provided by the sensor to digital information handled by the system. This forms the basis of what we call “smart sensors”, in which a large group of resistive sensors transforms the measurement of a certain physical magnitude in the variation of the value of a resistor [1]. Thus, we use resistive sensors to measure temperature (known as thermistors), gas detection [2], or magnetoresistive sensors [3,4]. These sensors can also be grouped in arrays, for example in anemometry [5], and for gas detection [6] or in tactile piezoresistive sensor arrays [7,8]. Different methods can be used to perform resistance-to-digital conversion. One of the most popular methods, which performs this conversion without the need for analogue-to-digital converters (ADC) is known as the direct interface circuit (DIC). This method [9,10] requires a minimum number of components: the resistive sensor itself, some calibration resistors, and a capacitor. The minimal hardware used makes the method simple, easy to integrate into any system, and economical, achieving a performance similar to that of the ADCs [11]. In recent years, a number of papers have been published in which a DIC was used with a programmable digital device as a microcontroller [12,13,14,15] and field programmable gate array (FPGA) [16,17], which shows the versatility of the method. DICs are not only used for the measurement of resistive sensors, but are also used, according to the literature, for capacitive [18,19,20,21,22] and inductive sensors [23,24,25,26].

The main problem with a DIC is the time needed to convert information. The time varies depending on the version of DIC in question (the different versions will be analyzed in the next section). The fastest DIC uses the single-point calibration method (SPCM) [27], although it is also the one with the greatest error. The SPCM needs to charge and discharge a capacitor twice. Charging is always through a small resistor, or possibly even without it if the digital device so allows, therefore requiring a much-reduced time. However, discharges are made once through the resistor of the sensor, *R*, and once through a known calibration resistor. Simply making the quotient of these discharge times is enough to obtain the value of *R* (the discharge times are measured in cycles of the internal clock of the programmable logic device, or PDD). All the simple arithmetic operations needed in these DICs, or others, are performed internally in the PDD, and the time spent on them is negligible compared to that required for the capacitor charge and discharge processes. For this reason, the time required to obtain *R* can be approximated by the sum of the two charge and discharge times. In the most sophisticated and accurate versions of DIC, the two-point calibration method (TPCM), and the three-signal auto-calibration method (TSACM) [27], the conversion time is increased to three capacitor charge and discharge times, with discharges now taking place through the resistors to be measured and two calibration resistors.

A naïve idea to decrease the time needed to obtain *R* is to think that the problem could be solved by decreasing the capacity of the capacitor, *C*. However, [28] shows that the time constant in the capacitor discharge process must be greater than a certain value in order to achieve optimum performance in a DIC. This minimum value is dependent on the range of resistors to be measured, but also on the PDD used and on the circuit’s electrical and quantification noise.

The problem may be even more serious if we are not measuring an isolated resistive sensor, but rather, an array of resistive sensors—such as for example, in the case of tactile sensors or artificial noses. In these cases, reducing the measurement time of an array frame is essential in order to obtain the characteristics of the system it is interacting with in real time, e.g., to obtain information about grip or slippage in a tactile sensor [29] or to obtain instant information in an artificial nose.

In this article, we analyze the different types of DICs and demonstrate how the TPCM offers better performance when estimating the resistance of a sensor than the TSACM. However, as we have seen, the TPCM may require a long time to complete the necessary measurements. For this reason, a new method is developed to reduce the measurement time. This reduction is achieved without any modification in the structure of a TPCM conventional DIC, and therefore, without any hardware cost. Only the time measurements change, allowing conversion time reductions of up to 55%. The experimental results obtained with an FPGA and the new method show that the reduction in conversion time is achieved without modifying the accuracy of the measurements.

The structure of the paper is as follows. Section 2 shows the operating principles of the different types of DICs and their fundamental characteristics. Section 3 presents the first version of the improved DIC: Fast Calibration Method I. Section 4 sets out a new version of the DIC with different characteristics to the one presented in Section 3: Fast Calibration Method II. Section 5 presents the experimental results obtained for the proposed new methods. These results are also discussed and compared with the TPCM. Finally, the conclusions are presented in Section 6.

## 2. Operating Principles and Types of DIC

As indicated, all types of DIC are based on a comparison of discharge times: one of these times is obtained by discharging through a PDD pin attached to the resistor of the sensor we intend to measure, *R*, and the other times (which can be one or two) are obtained through pins connected to known calibration resistors, *R_ci_*. As mentioned in the introduction, the simplest DIC is the SPCM, as it uses only one calibration resistor. As a result, the time taken in the resistance-to-digital conversion is the shortest of all; however, the accuracy is much lower (the relative error of this method can be three orders of magnitude greater than that obtained by the other methods [30]), for which reason it is not normally used in practical applications, and was not studied during this research.

### TPCM Analysis

**Figure 1:**
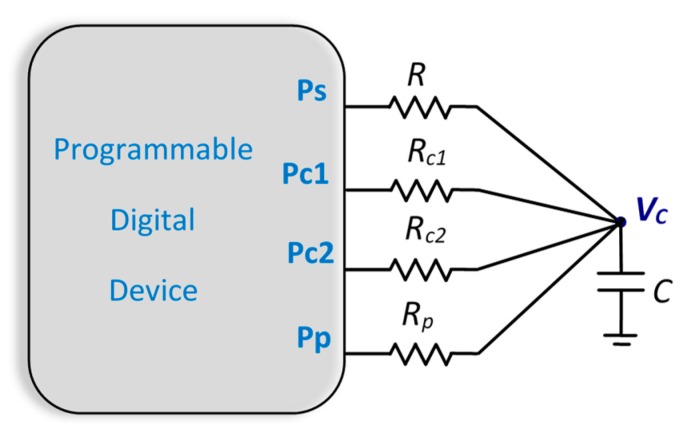
Circuit used in the two-point calibration method (TPCM).

The TPCM uses two calibration resistors, as shown in Figure 1. The pull-up resistor, *R_p_*, is used to charge *C* to the supply voltage of the PDD buffers (configuring the Pp pin as logic 1 output), and its value is as small as the PDD specifications allow, in order to reduce the charge time to the extent possible. It is also found in the literature [31] that the use of *R_p_* can reduce the influence of power-supply noise on circuit performance. To achieve this, the rest of the pins of Figure 1 can also be configured as logic 1 outputs. Then, a discharge process is performed through *R*, *R_c1_*, or *R_c2_* (regardless of order), configuring the appropriate pin (Ps, Pc1, or Pc2, respectively) as logic 0 output and keeping the other pins in a high impedance state, HZ, which is equivalent to configuring a pin as an input. The Pp pin is also configured as an input and is in charge of detecting the instant at which the capacitor voltage drops to a value considered logic 0 by the PDD. This succession of charge and discharge processes is carried out for the three resistors.

The discharge time, *T_R_*, through a resistor *R* is given by:

(1) $$T_{R}=\left(R+R_{o}\right)C\ln\left(\frac{V_{i}}{V_{f}}\right)$$

where *R_o_* is the output resistance of each pin configured as logic 0 output, *V_i_* is the initial discharge voltage (normally *V_DD_*), and *V_f_* is the final process voltage (the threshold voltage at which an input pin goes from detecting a logic 1 to a logic 0). Considering Equation (1), the TPCM uses the following equation to estimate the value of R:

(2) $$R=\frac{T_{R}-T_{Rc1}}{T_{Rc2}-T_{Rc1}}\left(R_{c2}-R_{c1}\right)+R_{c1}$$

where *T_Rc1_* and *T_Rc2_* are the discharge times through *R_c1_* and *R_c2_*, respectively. Given how the discharge times are used in Equation (2), we can eliminate the dependence of *R_o_*, *C*, and the natural logarithm that appears in Equation (1) when estimating *R*.

Two parameters can be taken to assess the speed of the resistance-to-digital conversion: the maximum time taken to discharge the capacitor to *V_f_*, *T_Rmax_*, and the maximum total time in the *R* measurement process, *T_max_*, which is the result of adding the time needed to evaluate *R_c1_* and *R_c2_* to *T_Rmax_*. We take these two parameters, as there may be applications in which a simultaneous calibration is not necessary for each *T_R_* measurement, but rather the calibration is performed at certain intervals between several *T_R_* measurements. In the case of the TPCM, these two parameters are given by:

(3) $$T_{max}(TPCM)=3\cdot T_{ch\arg e}+T_{R\max}+T_{Rc1}+T_{Rc2}$$

(4) $$T_{Rmax}(TPCM)=\left(R_{max}+R_{o}\right)C\ln\left(\frac{V_{DD}}{V_{f}}\right)$$

where *T_charge_* is the charge time (as commented earlier, the circuit is designed so that this is minimal, meaning that it is much smaller than the other times that appear in the member on the right of Equation (3)). If we also take into account that $R_{0}\ll R,R_{c1},R_{c2}$, we can approximate Equation (3) and Equation (4) by:

(5) $$T_{max}(TPCM)\approx k\cdot \left(R_{\max}+R_{c1}+R_{c2}\right)$$

(6) $$T_{Rmax}(TPCM)\approx k\cdot R_{max}$$

where *k* is a constant for each circuit:

(7) $$k=C\ln\left(\frac{V_{DD}}{V_{f}}\right)$$

Moreover, positioning *R_c1_* in 15% of the range between the maximum and minimum resistance to be measured (Δ*R*) is established as the optimum design criteria for the TPCM in [32]. In this reference, it also is found that *R_c2_* must be in 85% of the range. Therefore, *T_max_(TPCM)* is given by:

(8) $$T_{max}(TPCM)\approx k\cdot \left(2R_{max}+R_{min}\right)$$

As *k* is a characteristic of each circuit (we assume that a minimum value of *C* has been used according to [27]) and *R_max_* and *R_min_* are determined by the type of sensor, there is, initially, no option to reduce this time. Finally, Equation (8) can also be written as:

(9) $$T_{max}(TPCM)\approx \left(2+\frac{R_{min}}{R_{max}}\right)T_{Rmax}(TPCM)$$

which, in the very common case that $R_{\max}\gg R_{\min}$ will be:

(10) $$T_{max}(TPCM)\approx 2\cdot T_{Rmax}(TPCM)$$

**Figure 2:**
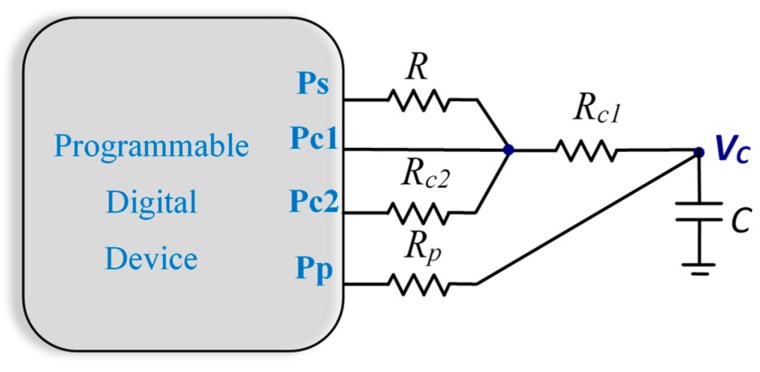
Circuit used in the three-signal auto-calibration method (TSACM).

The third type of DIC is the TSACM, the circuit for which is shown in Figure 2. This method was proposed [33] for capacitance measurement, but works in the same way for resistances.

As in the TPCM, this circuit measures three discharge times: *T_R+Rc1_*, the discharge time through resistors *R* and *R_c1_* positioned serially, *T_Rc2+Rc1_*, the discharge time through resistors *R_c1_* and *R_c2_* positioned serially, and *T_Rc1_*. With these times and Equation (1), we can find the value of *R* as:

(11) $$R=\frac{T_{R+Rc1}-T_{Rc1}}{T_{Rc2+Rc1}-T_{Rc1}}R_{c2}$$

Equation (11) is simpler to evaluate than Equation (2), although the difference in terms of time and hardware in current PDDs can be insignificant. To assess the maximum conversion times, it must be remembered that, maintaining the criteria for the calibration resistor values indicated above, *R_c1_* will be found around 15% of the measurement range, while *R_c2_+R_c1_* (which now plays the *R_c2_* role in the TPCM) will be 85%, meaning that *R_c2_* will be in 70% of the measurement range. Considering this, the maximum discharge time with this method, *T_Rmax_(TSACM)*, will be given, for wide ranges, by:

(12) $$T_{Rmax}(TSACM)\approx 1.15\cdot k\cdot R_{max}=1.15\cdot T_{Rmax}(TPCM)$$

Likewise, *T_max_* for this method, *T_max_(TSACM)*, will be:

(13) $$T_{max}(TSACM)\approx k\cdot \left(R_{max}+3R_{c1}+R_{c2}\right)=k\cdot \left(2.15\cdot R_{max}+1.85R_{min}\right)$$

Using Equation (8), this last equation is transformed into:

(14) $$T_{max}(TSACM)\approx T_{max}(TPCM)+T_{Rc1}$$

Equations (12) and (14) show that obtaining resistance values always requires more time in the TSACM than in the TPCM.

Another drawback of the TSACM when compared to TPCM is that, as shown in [27], the uncertainty in discharge time measurements is linearly related to the value of the resistor to be measured, with the slope of the line being positive. This means that $u\left(T_{R+Rc1}\right)>u\left(T_{R}\right)$ and $u\left(T_{Rc2+Rc1}\right)>u\left(T_{Rc2}\right)$. Considering this fact and applying the law of propagation of uncertainties [34], the variance of *R* using TPCM, $u_{TPCM}^{2}\left(R\right)$, is:

(15) $$\begin{array}{c} u_{TPCM}^{2}(R)={\left(\frac{\partial R}{\partial T_{R}}\right)}^{2}u^{2}(T_{R})+{\left(\frac{\partial R}{\partial T_{Rc1}}\right)}^{2}u^{2}(T_{Rc1})+{\left(\frac{\partial R}{\partial T_{Rc2}}\right)}^{2}u^{2}(T_{Rc2})\\ =\frac{{\left(R_{c2}-R_{c1}\right)}^{2}}{{\left(T_{Rc2}-T_{Rc1}\right)}^{2}}\left(u^{2}(T_{R})+{\left(\frac{T_{R}-T_{Rc2}}{T_{Rc2}-T_{Rc1}}\right)}^{2}u^{2}(T_{Rc1})+{\left(\frac{T_{R}-T_{Rc1}}{T_{Rc2}-T_{Rc1}}\right)}^{2}u^{2}(T_{Rc2})\right)\\ =\frac{1}{k^{2}}\left(u^{2}(T_{R})+{\left(\frac{R-R_{c2}}{R_{c2}-R_{c1}}\right)}^{2}u^{2}(T_{Rc1})+{\left(\frac{R-R_{c1}}{R_{c2}-R_{c1}}\right)}^{2}u^{2}(T_{Rc2})\right) \end{array}$$

while the variance of *R* using TSACM, $u_{TSACM}^{2}\left(R\right)$, is:

(16) $$\begin{array}{c} u_{TSACM}^{2}(R)={\left(\frac{\partial R}{\partial T_{R+Rc1}}\right)}^{2}u^{2}(T_{R+Rc1})+{\left(\frac{\partial R}{\partial T_{Rc1}}\right)}^{2}u^{2}(T_{Rc1})+{\left(\frac{\partial R}{\partial T_{Rc2+Rc1}}\right)}^{2}u^{2}(T_{Rc2+Rc1})=\\ \frac{R_{c2}^{2}}{{\left(T_{Rc2+Rc1}-T_{Rc1}\right)}^{2}}\left(u^{2}(T_{R+Rc1})+{\left(\frac{T_{R+Rc1}-T_{Rc2+Rc1}}{T_{Rc2+Rc1}-T_{Rc1}}\right)}^{2}u^{2}(T_{Rc1})+{\left(\frac{T_{R+Rc1}-T_{Rc1}}{T_{Rc2+Rc1}-T_{Rc1}}\right)}^{2}u^{2}(T_{Rc2+Rc1})\right)\\ =\frac{1}{k^{2}}\left(u^{2}(T_{R+Rc1})+{\left(\frac{R-R_{c2}}{R_{c2}}\right)}^{2}u^{2}(T_{Rc1})+{\left(\frac{R}{R_{c2}}\right)}^{2}u^{2}(T_{Rc2+Rc1})\right) \end{array}$$

Considering that, in Equation (15), *R_c2_* – *R_c1_* is equal to *R_c2_* in Equation (16) (if its values continue to maintain the criteria outlined above), it will be fulfilled that:

(17) $$u_{TPCM}(R)<u_{TSACM}(R)$$

and, therefore, the estimates of *R* in the TSACM will be less accurate than in the TPCM.

Based on these results, it is evident that the small computational advantage of using Equation (11) rather than Equation (2) does not compensate for the drawbacks arising from the TSACM needing more time to estimate *R*, and the accuracy of the measurements is also lower. Therefore, the TPCM should be the preferred method for a DIC.

However, the main drawback of the TPCM is the time needed to estimate *R*. According to Equation (6), *T_Rmax_* is proportional to *R_max_* and, depending on the sensor, this value can be very high, meaning that the temporal performance of the DIC may be insufficient in certain applications, as explained in the Introduction. A new measurement methodology based on the TPCM and using the same DIC is developed with the aim of reducing *T_Rmax_* and *T_max_*. We call this new methodology the Fast Calibration Method (FCM), and it also needs three charge and discharge processes. Depending on how discharge times are reduced, the new methodology has two versions, FCM I and FCM II, as presented below.

## 3. Fast Calibration Method I

The basic idea of FCM I is to use the minimum calibration resistor, *R_c1_*, to speed up the discharge process through *R*, when necessary. The way to proceed is as follows: the charge and discharge processes alternate as in the TPCM, and the *R_c1_* and *R_c2_* discharge processes are carried out in the same way. Similarly, if the discharge time through *R*, *T_R_*, is less than a certain value, *T_x_* (selected by the designer), discharge takes place as in the TPCM. Then, Equation (2) can still be used to estimate the value of *R*, and Equations (5) and (6) can be used to evaluate *T_max_* and *T_Rmax_*, respectively. However, if a logic 0 has not been detected in Pp for time *T_x_* after the discharge through *R* began (this condition is equivalent to *T_R_* > *T_x_*), the Ps pin is configured as *HZ*, and the discharge continues through *R_c1_*. This constitutes what we will call the modified discharge procedure. The only conditions that *T_x_* must fulfill are:

(18) $$T_{Rc1}<T_{x}<T_{Rmax}$$

These conditions come from the fact that, in order for the method to be consistent, it must be verified that *T_R_* > *T_x_* and that, in order for a time reduction in *T_R_* to be achieved, it must obviously be the case that *T_x_* < *T_Rmax_*.

**Figure 3:**
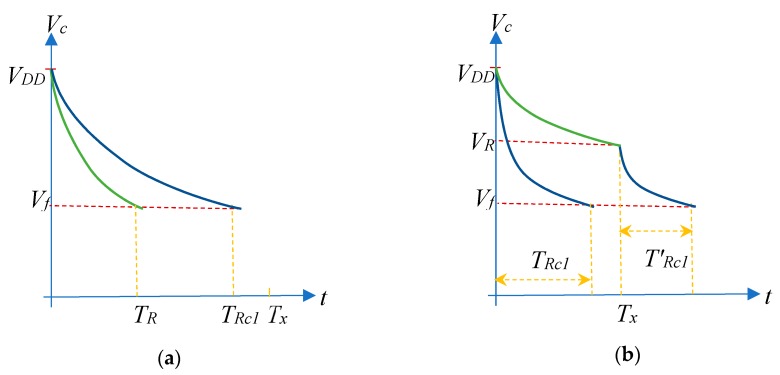
Evolution of capacitor voltage, *V_c_*, in discharges through *R_c1_* (blue) and *R* (green). *Vc(t)* will vary in accordance with the comparison of the value of the discharge time through *R*, *T_R_*, with the constant *T_x_*. (**a**) Situation in which *T_R_* is less than *T_x_*. (**b**) Situation in which *T_R_* is greater than *T_x_*.

Both discharge processes are shown in Figure 3. The situation that appears in Figure 3a does not require further attention, as it coincides with the steps to be performed in the TPCM. However, in the situation illustrated by Figure 3b: *T_x_* < *T_Rmax_* and *R* cannot be estimated using Equation (2). Nevertheless, it is possible to find a procedure to find *R*, as will be shown below.

As shown in Figure 3b, when discharging through *R*, the voltage in the capacitor will have reached value *V_R_* once time *T_x_* has passed, which will obviously be a function of *R* (*V_R_* is greater than the logic 0 threshold voltage of the PDD input pins, *V_f_*). *T_x_* can be expressed as:

(19) $$T_{x}=\left(R+R_{o}\right)C\ln\left(\frac{V_{DD}}{V_{R}}\right)$$

Moreover, if we define $T_{Rc1}^{\prime }(R)$ as the time taken by *R_c1_* to discharge the capacitor from *V_R_* to *V_f_*, this time is given by:

(20) $$T_{Rc1}^{\prime }(R)=\left(R_{c1}+R_{o}\right)C\ln\left(\frac{V_{R}}{V_{f}}\right)$$

Using the values of *T_Rc1_* and *T_Rc2_* given by Equation (1), *T_x_* can also be written as:

(21) $$\begin{array}{c} T_{x}=\left(R+R_{o}\right)C\ln\left(\frac{V_{DD}}{V_{R}}\right)=\left(R+R_{o}\right)C\ln\left(\frac{V_{DD}}{V_{f}}\right)\frac{\ln\left(\frac{V_{DD}}{V_{f}}\right)-\ln\left(\frac{V_{R}}{V_{f}}\right)}{\ln\left(\frac{V_{DD}}{V_{f}}\right)}\\ =\left(R+R_{o}\right)C\ln\left(\frac{V_{DD}}{V_{f}}\right)\cdot \left(\frac{T_{Rc1}-T_{Rc1}^{\prime }(R)}{T_{Rc1}}\right) \end{array}$$

and operating with this expression, we find that:

(22) $$\frac{R-R_{c1}}{R_{c2}-R_{c1}}=\frac{\left(R+R_{o}\right)C\ln\left(\frac{V_{DD}}{V_{f}}\right)-\left(R_{c1}+R_{o}\right)C\ln\left(\frac{V_{DD}}{V_{f}}\right)}{\left(R_{c2}+R_{o}\right)C\ln\left(\frac{V_{DD}}{V_{f}}\right)-\left(R_{c1}+R_{o}\right)C\ln\left(\frac{V_{DD}}{V_{f}}\right)}=\frac{\frac{T_{Rc1}\cdot T_{x}}{T_{Rc1}-T_{Rc1}^{\prime }(R)}-T_{Rc1}}{T_{Rc2}-T_{Rc1}}$$

finally resulting in:

(23) $$R=\frac{\frac{T_{Rc1}\cdot T_{x}}{T_{Rc1}-T_{Rc1}^{\prime }(R)}-T_{Rc1}}{T_{Rc2}-T_{Rc1}}\left(R_{c2}-R_{c1}\right)+R_{c1}$$

Therefore, *R* is estimated by FCM I according to the following pair of equations and conditions:

(24) $$R=\{\begin{array}{cc} \frac{T_{R}-T_{Rc1}}{T_{Rc2}-T_{Rc1}}\left(R_{c2}-R_{c1}\right)+R_{c1}, & T_{R}<T_{x}\\ \frac{{\frac{T_{Rc1}\cdot T_{x}}{T_{Rc1}-T_{Rc1}^{\prime }(R)}}_{x}-T_{Rc1}}{T_{Rc2}-T_{Rc1}}\left(R_{c2}-R_{c1}\right)+R_{c1}, & T_{R}\geq T_{x} \end{array}$$

Expression (23) is slightly more complex than Equation (2) due to the computational cost of a division, a subtraction, and an extra multiplication, as well as the comparison between times required to decide which estimate to use. However, below, it is shown that Equation (23) allows a significant reduction in *T_Rmax_* and, in consequence, also in *T_max_*.

In order to find *T_Rmax_* in this calibration method, it must be taken into account that Equations (5) and (6) are still valid if *T_R_* < *T_x_*. If the calibration resistors and the charge time are identical in FCM I and the TPCM, the only difference appears in *T_R_* when *T_R_* > *T_x_*. For convenience, *T_x_* and $T_{Rc1}^{\prime }$ will be expressed as a function of a parameter *α*, such that:

(25) $$T_{x}=\alpha \cdot T_{Rmax}(TPCM)\approx \alpha \cdot k\cdot R_{max}$$

with $T_{Rc1}/T_{Rmax}<\alpha <1$ (the condition α<1 obviously means that $T_{x}<T_{R\max}$ must be fulfilled).

Moreover, the maximum value of *V_R_*, *V_Rmax_*, occurs when discharging through *R_max_* for time *T_x_*

(26) $$V_{Rmax}=V_{DD}e^{\frac{-\alpha T_{Rmax}(TPCM)}{\left(R_{max}+R_{o}\right)\cdot C}}$$

and, therefore, the maximum of $T_{Rc1}^{\prime }(R)$, $T_{Rc1}^{\prime }(R_{max})$, would be given by:

(27) $$\begin{array}{c} T_{Rc1}^{\prime }(R_{max})=\left(R_{c1}+R_{o}\right)C\ln\left(\frac{V_{Rmax}}{V_{f}}\right)=\left(R_{c1}+R_{o}\right)C\left[\ln\left(\frac{V_{DD}}{V_{f}}\right)-\frac{\alpha \cdot T_{Rmax}(TPCM)}{\left(R_{max}+R_{o}\right)\cdot C}\right]\\ =T_{Rc1}(R)-\frac{\alpha \cdot T_{Rmax}(TPCM)\cdot \left(R_{c1}+R_{o}\right)}{\left(R_{max}+R_{o}\right)}\approx kR_{c1}-\alpha kR_{c1}=(1-\alpha )kR_{c1} \end{array}$$

where it has again been considered that *R_o_* is negligible in comparison with the other resistors that appear in Equation (27). With this result:

(28) $$T_{Rmax}(FCM\;I)=T_{x}+T_{Rc1}^{\prime }(R_{max})=\alpha \cdot k\cdot R_{max}+(1-\alpha )\cdot k\cdot R_{c1}$$

and bearing in mind Equation (6), we can write:

(29) $$T_{Rmax}(FCM\;I)=T_{Rmax}(TPCM)-\left(1-\alpha \right)\cdot k\cdot \left(R_{max}-R_{c1}\right)$$

Hence, as the second term on the right of this equation is always greater than zero, it is verified that $T_{Rmax}(FCM\;I)<T_{Rmax}(TPCM)$. Since *T_Rc1_* and *T_Rc2_* are the same for both methods, we also have:

(30) $$T_{max}(FCM\;I)=T_{max}(TPCM)-\left(1-\alpha \right)\cdot k\cdot \left(R_{max}-R_{c1}\right)$$

and therefore, also $T_{max}(FCM\;I)<T_{max}(TPCM)$. For example, if we again consider $R_{c1}=0.15\cdot \Delta R+R_{min}$ and $R_{c2}=0.85\cdot \Delta R+R_{min}$, we find that:

(31) $$T_{Rmax}(FCM\;I)=\left[\left(0.15+0.85\cdot \alpha \right)+\frac{0.85\cdot \left(1-\alpha \right)R_{min}}{R_{max}}\right]T_{Rmax}(TPCM)$$

and with $R_{\max}\gg R_{\min}$:

(32) $$T_{Rmax}(FCM\;I)\approx \left(0.15+0.85\cdot \alpha \right)\cdot T_{Rmax}(TPCM)$$

Moreover, with the same choices and approximations, we have:

(33) $$T_{max}(FCM\;I)=\left(0.575+0.425\cdot \alpha \right)\cdot T_{max}(TPCM)$$

According to Equations (29) and (30), the reduction in time will depend on *α*. Hence, the smaller this parameter, the greater the reduction in measurement times. However, *α* has an influence on the precision and accuracy of the *R* estimates, as will be analyzed below, meaning that there is a trade-off between method speed and accuracy.

We know that the uncertainty of FCM I in estimating the value of *R*, *u_FCM I_(R)*, is equal to *u_TPCM_(R)* if *T_R_* < *T_x_*. The differences between the two uncertainties appear if *T_R_* ≥ *T_x_*. In this case, the variance in the measurements for the FCM I, $u_{FCM\;I}^{2}\left(R\right)$, is given by:

(34) $$u_{FCM\;I}^{2}(R)={\left(\frac{\partial R}{\partial T_{Rc1}^{\prime }(R)}\right)}^{2}u^{2}(T_{Rc1}^{\prime }(R))+{\left(\frac{\partial R}{\partial T_{Rc1}}\right)}^{2}u^{2}(T_{Rc1})+{\left(\frac{\partial R}{\partial T_{Rc2}}\right)}^{2}u^{2}(T_{Rc2})$$

which we have calculated using the value of *R* given by Equation (23). Moreover, when equating Equations (2) and (23), we have:

(35) $$T_{R}=\frac{T_{Rc1}}{T_{Rc1}-T_{Rc1}^{\prime }(R)}T_{x}$$

Using Equations (23) and (35) to evaluate Equation (34), after a few simple calculations, we obtain:

(36) $$u_{FCM\;I}^{2}(R)=\frac{1}{k^{2}}\left[{\left(\frac{T_{R}^{2}}{T_{x}T_{Rc1}}\right)}^{2}u^{2}(T_{Rc1}^{\prime }(R))+{\left(\frac{R-R_{c2}}{R_{c2}-R_{c1}}-\frac{T_{R}}{T_{Rc1}}\cdot \left(\frac{T_{R}}{T_{x}}-1\right)\right)}^{2}u^{2}(T_{Rc1})+{\left(\frac{R-R_{c1}}{R_{c2}-R_{c1}}\right)}^{2}u^{2}(T_{Rc2})\right]$$

Comparing this result to Equation (15) shows that the contribution of the variance due to *T_Rc2_* is identical in both equations. Moreover, we can find the relationship between $u^{2}(T_{Rc1}^{\prime }(R))$ and *u^2^(T_R_)* because, as indicated in [27], the uncertainties in discharge time measurements are approximately proportional to the discharge resistance value when the trigger event occurs (if quantification error is neglected):

(37) $$u\left(T_{R}\right)\approx \frac{\varepsilon }{V_{f}\cdot \ln\left(\frac{V_{i}}{V_{f}}\right)}\cdot T_{R}\approx \frac{\varepsilon }{V_{f}}CR$$

where *ε* is related to circuit noise. With this result, we can write:

(38) $$\frac{u\left(T_{Rc1}^{\prime }(R)\right)}{u\left(T_{R}\right)}\approx \frac{\ln\left(\frac{V_{i}}{V_{f}}\right)}{\ln\left(\frac{V_{R}}{V_{f}}\right)}\cdot \frac{T_{Rc1}^{\prime }(R)}{T_{R}}=\frac{T_{Rc1}}{T_{Rc1}^{\prime }(R)}\cdot \frac{T_{Rc1}^{\prime }(R)}{T_{R}}=\frac{T_{Rc1}}{T_{R}}$$

and also:

(39) $$\frac{u\left(T_{Rc1}^{\prime }(R)\right)}{u\left(T_{R}\right)}\approx \frac{R_{c1}}{R}$$

Considering Equation (38), it is verified that:

(40) $$\frac{{\left(\frac{T_{R}^{2}}{T_{x}T_{Rc1}}\right)}^{2}u^{2}(T_{Rc1}^{\prime }(R))}{u^{2}(T_{R})}\approx {\left(\frac{T_{R}}{T_{x}}\right)}^{2}>1$$

Thus, the addend due to the variance of $T_{Rc1}^{\prime }(R)$ in Equation (36) is always greater than that of the variance of *T_R_* in Equation (15).

Finally, as the addend due to the variance of *T_Rc1_* in the FCM I is greater than its equivalent in the TPCM whenever $R<R_{c2}$, we can conclude that, in this situation, $u_{FCM\;I}^{2}(R)>u_{TPCM}^{2}(R)$ and furthermore, a decrease of *T_x_* (i.e. *α*) always means an increase in $u_{FCM\;I}^{2}(R)$. However, if $R\geq R_{c2}$, a simple relationship between $u_{FCM\;I}^{2}(R)$ and $u_{TPCM}^{2}(R)$ cannot be extracted. This relationship will depend on each specific value of *T_x_* and *R*.

One last question remains to be analyzed regarding this method: the *R_c2_* measurement. For all the foregoing, it is obvious that *R_c2_* discharges the capacitor up to *V_f_* if $T_{Rc2}<T_{x}$. However, even if $T_{Rc2}>T_{x}$, the complete discharge process is also carried out through this resistor in FCM I. However, it is possible to perform the modified discharge process of *R_c2_* if $T_{Rc2}>T_{x}$. In this way, we achieve an additional reduction in *T_max_*, which is especially important in applications where a calibration in each reading of *R* is necessary.

## 4. Fast Calibration Method II

The basic idea of the Fast Calibration Method II (FCM II) is to further reduce $T_{max}(FCM\;I)$ by decreasing *T_Rc2_* using the modified discharge procedure for *R_c2_*. In order to do this, *T_x_* must fulfill:

(41) $$T_{Rc1}<T_{x}<T_{Rc2}$$

If this condition is fulfilled, the capacitor voltage is *V_Rc2_* in *T_x_* when discharging through *R_c2_*, and the time used in the discharge, from this voltage to *V_f_*, would be $T_{Rc1}^{\prime }(R_{c2})$. Following the reasoning used to find Equation (23), it can be deduced that when verifying Equation (41) and $T_{R}>T_{x}$, *R* will be given by:

(42) $$R=\frac{\frac{T_{Rc1}\cdot T_{x}}{T_{Rc1}-T_{Rc1}^{\prime }(R)}-T_{Rc1}}{\frac{T_{Rc1}\cdot T_{x}}{T_{Rc1}-T_{Rc1}^{\prime }(R_{c2})}-T_{Rc1}}\left(R_{c2}-R_{c1}\right)+R_{c1}=\frac{\frac{T_{x}}{T_{Rc1}-T_{Rc1}^{\prime }(R)}-1}{\frac{T_{x}}{T_{Rc1}-T_{Rc1}^{\prime }(R_{c2})}-1}\left(R_{c2}-R_{c1}\right)+R_{c1}$$

which can also be written as:

(43) $$R=\frac{T_{Rc1}-T_{Rc1}^{\prime }(R_{c2})}{T_{Rc1}-T_{Rc1}^{\prime }(R)}\cdot \frac{T_{x}+T_{Rc1}^{\prime }(R)-T_{Rc1}}{T_{x}+T_{Rc1}^{\prime }(R_{c2})-T_{Rc1}}\left(R_{c2}-R_{c1}\right)+R_{c1}$$

However, if $T_{R}<T_{x}$, the modified discharge procedure only applies to *R_c2_*, and therefore:

(44) $$R=\frac{T_{R}-T_{Rc1}}{\frac{T_{Rc1}\cdot T_{x}}{T_{Rc1}-T_{Rc1}^{\prime }(R_{c2})}-T_{Rc1}}\left(R_{c2}-R_{c1}\right)+R_{c1}$$

Equation (43) is more suitable than Equation (42) to find the value of *R,* since the sums of *T_x_* and $T_{Rc1}^{\prime }(R)$ or *T_x_* and $T_{Rc1}^{\prime }(R_{c2})$ can be generated by a single counter in the PDD simply without resetting the counter when *T_x_* is reached. Taking this into account, the number of additional operations with regard to Equation (2) is only one division, one multiplication, and two subtractions (apart from making the comparison between *T_R_* and *T_x_*). Moreover, the number of operations in Equation (44) is the same as in FCM I.

As for the temporal response, for *T_Rmax_(FCM II)*, we have:

(45) $$T_{Rmax}\left(FCM\;II\right)=T_{Rmax}\left(FCM\;I\right)$$

However, *T_max_(FCM II)* will be given by:

(46) $$T_{max}(FCM\;II)\approx 2T_{x}+T_{Rc1}^{\prime }(R_{max})+T_{Rc1}^{\prime }(R_{c2})+T_{Rc1}$$

Only $T_{Rc1}^{\prime }(R_{c2})$ needs to be analyzed to evaluate this expression, since the other terms are known. If we continue to use Equation (25) and proceed in the same way as when obtaining Equation (27), $T_{Rc1}^{\prime }(R_{c2})$ will be given by:

(47) $$\begin{array}{c} T_{Rc1}^{\prime }(R_{c2})=\left(R_{c1}+R_{o}\right)C\ln\left(\frac{V_{Rc2}}{V_{f}}\right)=\left(R_{c1}+R_{o}\right)C\left[\ln\left(\frac{V_{DD}}{V_{f}}\right)-\frac{\alpha \cdot T_{Rmax}(TPCM)}{\left(R_{c2}+R_{o}\right)\cdot C}\right]=\\ =T_{Rc1}(R)-\frac{\alpha \cdot T_{Rmax}(TPCM)\cdot \left(R_{c1}+R_{o}\right)}{\left(R_{c2}+R_{o}\right)}\approx kR_{c1}-\alpha kR_{max}\frac{R_{c1}}{R_{c2}} \end{array}$$

where *α* is still determined by Equation (25), and it is also necessary to fulfill:

(48) $$\frac{T_{Rc1}}{T_{Rmax}}<\alpha <\frac{T_{Rc2}}{T_{Rmax}}$$

Considering Equation (47), Equation (46) can be written as:

(49) $$\begin{array}{c} T_{max}\left(FCM\;II\right)\approx 2\cdot \alpha \cdot k\cdot R_{max}+k\cdot (1-\alpha )\cdot R_{c1}+k\cdot (1-\alpha \frac{R_{max}}{R_{c2}})\cdot R_{c1}+k\cdot R_{c1}=\\ =2\cdot \alpha \cdot k\cdot R_{max}+k\cdot \left[3-\alpha \left(1+\frac{R_{max}}{R_{c2}}\right)\right]\cdot R_{c1} \end{array}$$

We can also find the difference between *T_max_(FCM I)* and *T_max_(FCM II)*:

(50) $$T_{max}\left(FCM\;I\right)-T_{max}\left(FCM\;II\right)=T_{Rc2}-\left(T_{x}+T_{Rc1}^{\prime }(R_{c2})\right)=k\left(R_{c2}-R_{c1}\right)\left(1-\alpha \frac{R_{max}}{R_{c2}}\right)$$

considering the upper limit of *α* provided by Equation (48), the result of Equation (50) is always positive, meaning that $T_{max}\left(FCM\;II\right)<T_{max}\left(FCM\;I\right)$.

Moreover, if we use $R_{c1}=0.15\cdot \Delta R+R_{min}$, $R_{c2}=0.85\cdot \Delta R+R_{min}$ and $R_{\max}\gg R_{\min}$ again, we obtain:

(51) $$T_{max}\left(FCM\;II\right)\approx k\left(0.45+1.674\cdot \alpha \right)R_{max}=\left(0.225+0.837\cdot \alpha \right)T_{max}\left(TPCM\right)$$

To finish the study of this method, the uncertainty in estimating the value of *R*, *u_FCM II_(R)*, will be analyzed, as in the previous calibration methods, by evaluating variance $u_{FCM\;II}^{2}(R)$ according to the law of propagation of uncertainties applied to Equations (42) and (44):

(52) $$u_{FCM\;II}^{2}(R)={\left(\frac{\partial R}{\partial T_{Rc1}}\right)}^{2}u^{2}(T_{Rc1})+{\left(\frac{\partial R}{\partial T_{Rc1}^{\prime }(R)}\right)}^{2}u^{2}(T_{Rc1}^{\prime }(R))+{\left(\frac{\partial R}{\partial T_{Rc1}^{\prime }(R_{c2})}\right)}^{2}u^{2}(T_{Rc1}^{\prime }(R_{c2}))$$

For $T_{R}>T_{x}$, using Equation (42), making the partial derivatives, taking into account Equation (35) and also considering that for this case:

(53) $$T_{Rc2}=\frac{T_{Rc1}}{T_{Rc1}-T_{Rc1}^{\prime }(R_{c2})}T_{x}$$

obtains:

(54) $$u_{FCM\;II}^{2}(R)=\frac{1}{k^{2}}\left[{\left(\frac{R-R_{c2}}{R_{c2}-R_{c1}}\frac{T_{R}+T_{Rc2}}{T_{x}}\right)}^{2}u^{2}(T_{Rc1})+{\left(\frac{T_{R}^{2}}{T_{x}T_{Rc1}}\right)}^{2}u^{2}(T_{Rc1}^{\prime }(R))+{\left(\frac{R-R_{c1}}{R_{c2}-R_{c1}}\frac{T_{Rc2}^{2}}{T_{Rc1}T_{x}}\right)}^{2}u^{2}(T_{Rc1}^{\prime }(R_{c2}))\right]$$

Carrying out a study for this equation similar to the one for comparison $u_{TPCM}^{2}(R)$ and $u_{FCM\;I}^{2}(R)$, Equations (15) and (36), finds that

(55) $$T_{R}>\sqrt{T_{x}\cdot T_{Rc1}}\Rightarrow u_{FCM\;II}(R)>u_{TPCM}(R)$$

but if $T_{R}>T_{x}$, taking into account that $T_{Rc1}<T_{x}$, the condition in Equation (55) is always verified.

Moreover, if $T_{R}<T_{x}$, the law of propagation of uncertainty applied to Equation (44) generates the following result:

(56) $$u_{FCM\;II}^{2}(R)=\frac{1}{k^{2}}\left[u^{2}(T_{R})+{\left(\frac{T_{R}}{T_{Rc1}}\right)}^{2}u^{2}(T_{Rc1})+{\left(\frac{R-R_{c1}}{R_{c2}-R_{c1}}\frac{T_{Rc2}^{2}}{T_{Rc1}T_{x}}\right)}^{2}u^{2}(T_{Rc1}^{\prime }(R_{c2}))\right]$$

The only information provided by this equation is that if $T_{Rc1}<T_{R}<T_{x}$, then $u_{FCM\;II}(R)>u_{TPCM}(R)$. Based on the conclusions drawn from Equations (55) and (56), we can state that whenever $T_{Rc1}<T_{R}$, it is verified that $u_{FCM\;II}(R)>u_{TPCM}(R)$. In contrast, if $T_{R}<T_{Rc1}$, there is no clear relationship between the uncertainty of this method and the others. Finally, it is important to note that any decrease in *T_x_* in Equations (54) and (56) translates into an increase in $u_{FCM\;II}(R)$, meaning that this method again presents a trade-off between speed and accuracy. 

## 5. Materials and Methods

The two calibration methods proposed in this paper, FCM I and FCM II, were tested and compared with the traditional TPCM on an FPGA. The set-up for the circuit with the FPGA was made using a Xilinx Spartan3AN FPGA (XC3S50AN-4TQG144C) [35] with an operating frequency of 50 MHz. The time–digital conversion was performed by a 14-bit counter with a 20-ns time base. Using this counter allowed us to measure discharge times of up to 2^14^ clock cycles, 327.68 µs. A capacitor with a 47-nF rated value was selected, which also complies with the design rules proposed in [28]. Moreover, this FPGA works with independent supply voltages for the input/output blocks and the digital processing core, meaning that voltage noise due to digital processing is reduced. The voltage for the pins of the DIC was 3.3 V, and the maximum current that an output buffer of this FPGA could sink in order to maintain the integrity of the digital values was 24 mA. In order to demonstrate the generality of the proposed methods, some tests were also carried out with 12 mA as the maximum output buffer current. Finally, a battery of decoupling capacitors of different values was used in a position very close to the supply input pins. The printed circuit board where the circuit was mounted is an FR-4 fiberglass substrate with four layers, leaving internal layers for supply planes and external layers for the remaining signals.

Experimental tests were performed for 20 resistors with resistance values within the range of 260 Ω to 7500 Ω. The resistance values were chosen to clearly show the differences in the performances of the different calibration methods. This range was selected for two reasons. First, the range was wide enough to show the performance of the proposed methods and secondly, the range coincided with that of a resistive tactile sensor used by the authors. It was manufactured with a sheet of piezoresistive material by the company CIDETEC. This sheet had resistances of 7400 Ω for pressures of a few kPa up to 250 Ω for high pressures (around 280 kPa) [36]. In addition to the resistor to be measured, two additional calibration resistors were added in order to asses different calibration methods: *R_c1_* = 1098.1 Ω and *R_c2_* = 6165.3 Ω. All the resistors were measured using an Agilent 34401A digital multimeter. The measurements were repeated 500 times for every evaluation of the discharge time through each of the 20 resistors used in the tests. The discharge times through *R_c1_* and *R_c2_* were measured every time the measurement was repeated, meaning that 500 results could be obtained for *R*, each one with its own measurements. Therefore, the maximum errors in each of the methods were evaluated.

The logic circuits proposed in [37] were used to improve the detection of the trigger event in Pp. In essence, each circuit detected the same transition 1→0 in a slightly different way, in an attempt to reduce any influence on spurious transition measurements. This was finally achieved using the mean of the different detections.

## 6. Results and Discussion

**Figure 4:**
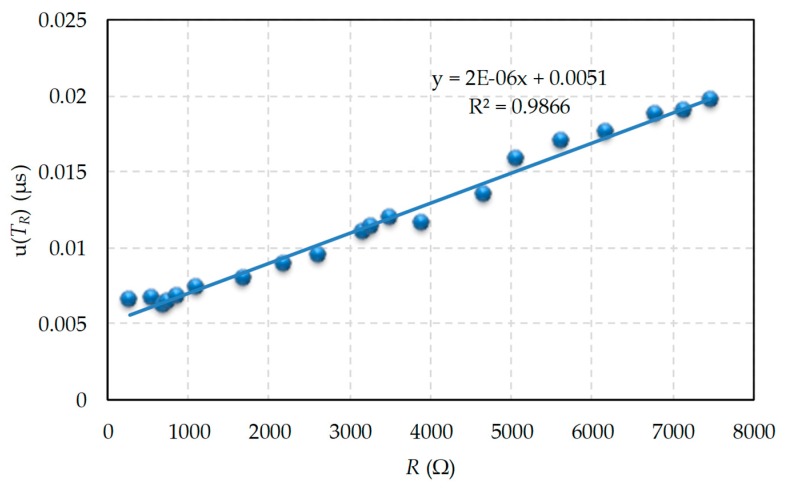
Uncertainty in the discharge time measurement (discharge takes place through a single resistor).

The experimental standard deviation for the discharge times of each resistor of the measured range were used as the uncertainty value. The uncertainties when the capacitor discharge was completed through the 20 resistors to be measured are shown in Figure 4. This chart confirms that there is a linear dependence between *u(T_R_)* and *R* (it should be remembered that the value of the capacitor was chosen to achieve this ratio). Moreover, the least square regression line equation, which appears in the same figure, shows that the independent term is small compared to the total value of *u(T_R_)*, as indicated in Equation (37), except for the smallest resistors.

**Figure 5:**
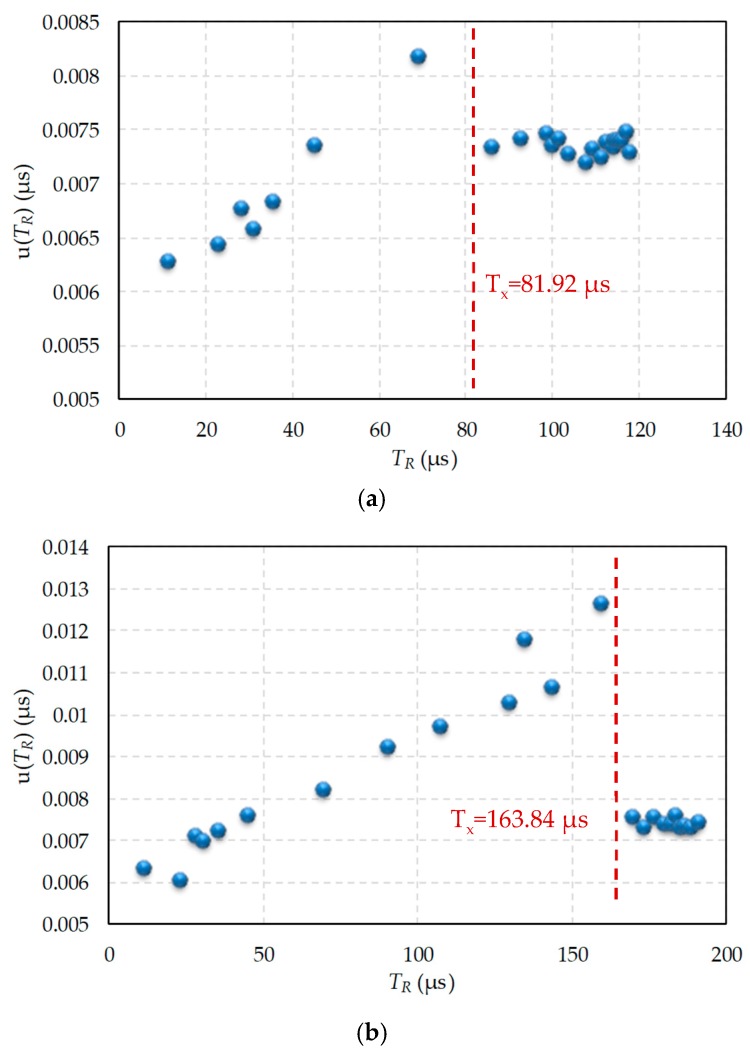

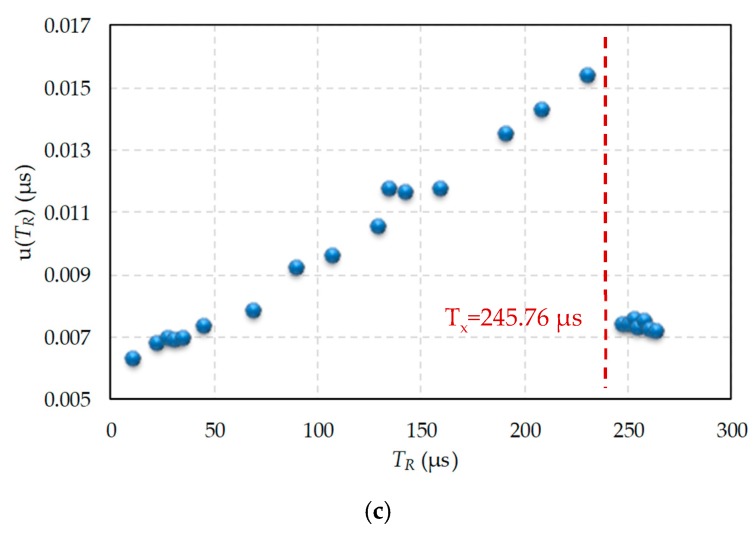
Uncertainty in the discharge time measurement when *R* intervenes, and the modified discharge procedure is applied. (**a**) *T_x_* = 81.92 µs, (**b**) *T_x_* = 163.84 µs, (**c**) *T_x_* = 245.76 µs.

Uncertainty does not behave in the same way in the case of discharge through the modified procedure with two resistors. Figure 5 shows uncertainty in the total discharge time measurement through *R* when the modified procedure was used. The value of T_x_ used in Figure 5b was half the maximum time we could measure with the FPGA counter, 163.84 µs, since this Tx needs to use only the most significant bit of the counter for its detection. For Figure 5a, T_x_ = 81.92 µs, the fourth part of the maximum value, since only the second most significant bit of the counter was needed for its detection. Finally, Figure 5c used T_x_ = 327.68 * 3/4 = 245.76 µs, so we only used the two most significant bits of the counter for detection. There were two clearly differentiated zones for the different *T_x_* values shown in Figure 5a,b,c: when discharge was solely through *R*, and when it was through *R* and *R_c1_*, respectively. In the first case, the results are those shown to the left of the vertical dashed red line that indicates the value of *T_x_*, and coincide with the results in Figure 4.

However, if the total discharge time is measured when *R* and *R_c1_* are used in the modified procedure, the time used to evaluate the uncertainty is $T_{x}+T_{Rc1}^{\prime }(R)$. The data now appear to the right of the vertical red line and uncertainty is practically constant, as the trigger event always occurred when discharging through *R_c1_*, which is consistent with Equation (37).

**Figure 6:**
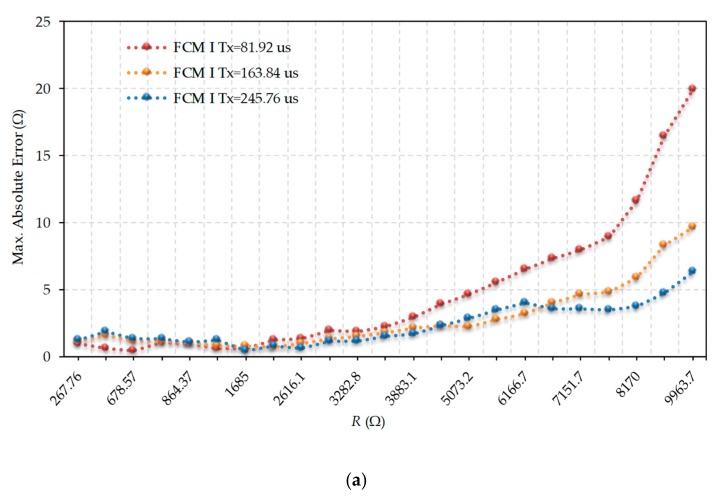

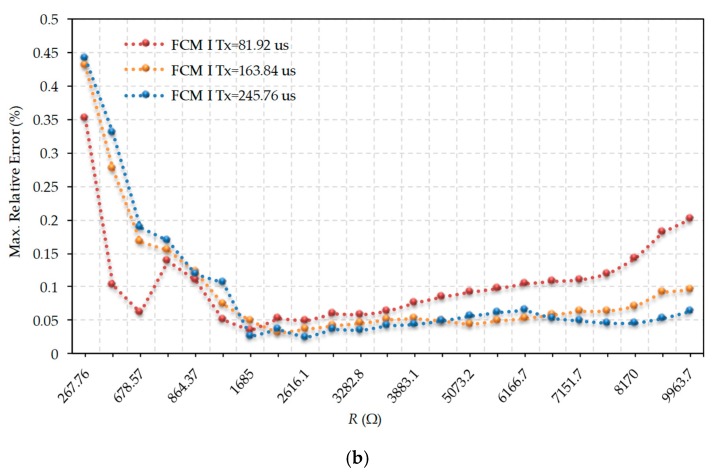
Maximum errors in the evaluation of *R* for different values of *T_x_* in Fast Calibration Method (FCM I). (**a**) Maximum absolute error, (**b**) Maximum relative error.

Figure 6 shows the errors obtained in estimating *R* when using FCM I. Figure 6a shows the maximum absolute errors, while Figure 6b shows the maximum relative errors. The errors were found for the three values of *T_x_* indicated above. For any *T_x_*, Figure 6 shows similar errors up to the 2198 Ω resistor. This is the case since *T_x_* = 81.92 µs was approximately the discharge time through a 2000 Ω resistor, with the modified discharge procedure applying only for higher values. As of this resistance value, the DIC with the lower *T_x_* started to show greater errors. However, the errors were very similar for *T_x_* = 163.84 µs and *T_x_* = 245.76 µs, even for high *R* values. This behavior suggests the possibility that, for each application, a range of values of *T_x_* that reduces the discharge time with minimal effect on accuracy can be found. Although the absolute errors increased whenever the resistance value to be measured increased, Figure 6b shows how the relative errors remained practically constant, as in the TPCM [27]. The maximum relative errors occurred for the smallest resistance values where the quantification error was greater (since it is independent of the value of *R*).

**Figure 7:**
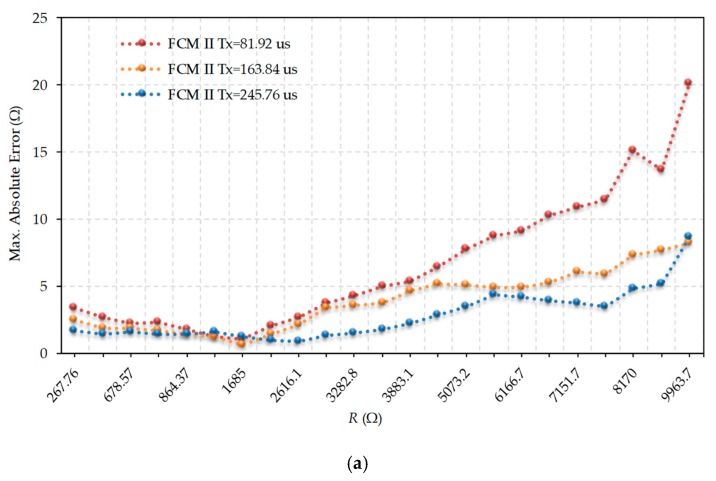

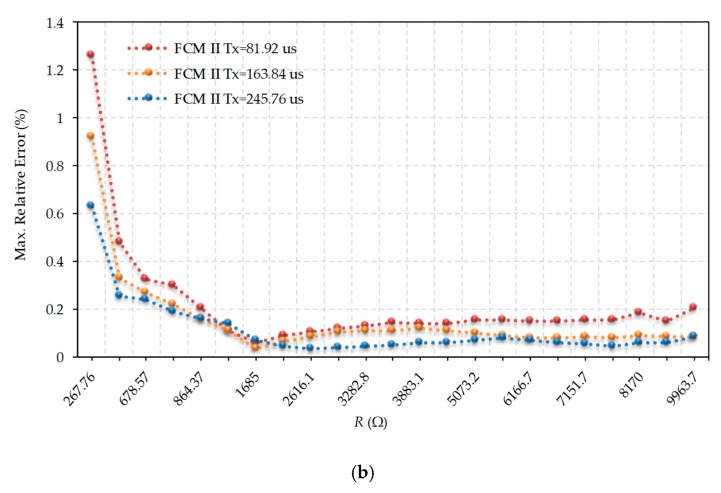
Maximum errors in the evaluation of *R* for different values of *T_x_* in Fast Calibration Method II (FCM II). (**a**) Maximum absolute error, (**b**) Maximum relative error.

Figure 7 shows the errors obtained in estimating *R* when using FCM II with the same three values of *T_x_* that were used in FCM I. It should be noted that these *T_x_* were always lower than *T_Rc2_* (which had a measured mean value of 253.3 µs), and therefore allowed the modified discharge procedure of *R_c2_*. For the same resistance values and *T_x_* values, the maximum errors shown in Figure 7 were always slightly larger than those in Figure 6, and maintained a fairly similar shape. Smaller *T_x_* had greater errors when the resistance values were above 2200 Ω.

**Figure 8:**
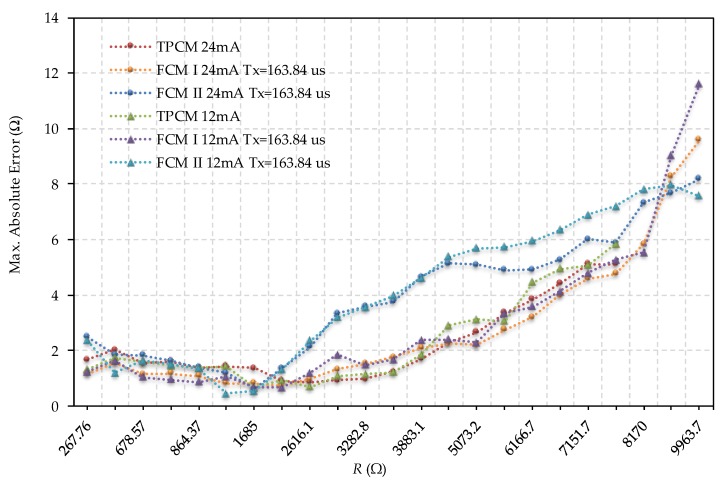

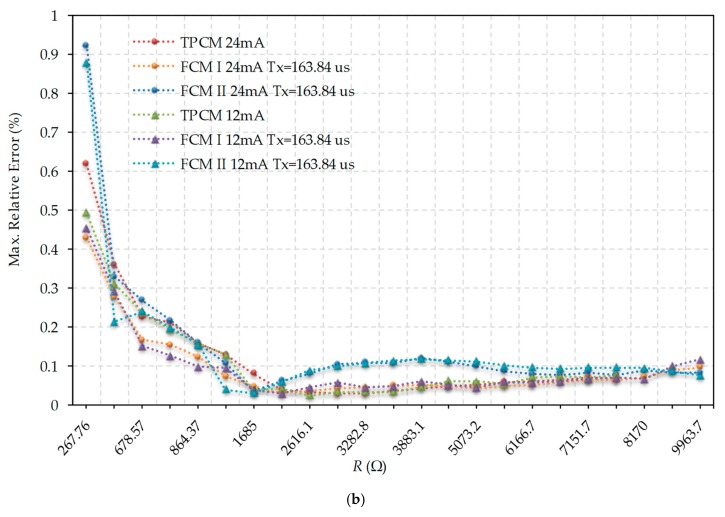
Comparison between errors evaluating *R* using the TPCM and the two FCMs for *T_x_* = 163.84 µs and for maximum output current configurations of 12 mA and 24 mA. (**a**) Comparison between the maximum absolute errors, (**b**) Comparison between the maximum relative errors.

Figure 8 shows the comparison of errors between the TPCM and the FCMs for *T_x_* = 163.84 µs and for maximum output currents of 12 mA and 24 mA. As can be seen, the shape of the error curves was similar in all methods and both output buffer configurations. FCM II presents the greatest errors if the resistance values were large, while TPCM and FCM I were very similar throughout the whole range. The relative errors of the three methods were also quite similar. The results shown in Figure 8 largely concur with the uncertainty study carried out for the FCMs. Another advantage of FCMs, as highlighted in Figure 8, is that greater resistance values can be measured in the two proposed FCMs for the maximum measurement time of the TPCM (which is determined by an internal FPGA counter). The maximum resistor for the TPCM is 7464.5 Ω, while for the FCMs, it is 9963.7 Ω).

The *R_max_* of the TPCM (7464.5 Ω) were used as a reference with regard to the reduction in time necessary to estimate the value of a resistor when using the FCMs. Table 1 shows the *T_Rmax_* and *T_max_* values for the different *T_x_* used in Figure 5, Figure 6 and Figure 7 in accordance with the method used. Obviously, the TPCM shows the same values independently of *T_x_*. Equally, *T_Rmax_* is the same value for the same *T_x_* in the two FCMs. However, *T_max_* is always lower in FCM II compared to the other methods. The reduction in *T_Rmax_* varies between 17–62%, while the reduction in *T_max_* varies between 9.5–55%, depending on the method used and the value of *T_x_*.

## 7. Conclusions

There are several direct interface circuit variants in the literature to convert the resistance of a sensor to digital information. These differ in the accuracy of the measurements, the time needed to perform the conversion, and the complexity of the arithmetic calculations. This article includes a study of these parameters that shows that, among the most accurate methods (two-point calibration method, or TPCM, and three-signal auto-calibration method, or TSACM), the TPCM is the most suitable choice, as it requires less time for conversion and also produces less uncertainty when estimating sensor resistance.

Despite being the fastest method, the TPCM requires three discharge times to measure a resistor. As the discharge time through the sensor’s resistor increases with the value of this resistor, this time can become excessive. To overcome this problem, we propose a modified discharge process in which part of the discharge (previously performed through the resistive sensor only) is performed with the smallest calibration resistor. This is what we call Fast Calibration Method I, (FCM I). If we also apply this modified discharge procedure to the higher calibration resistor, *R_C2_*, then we will be working with what we call Fast Calibration Method II (FCM II). Logically, FCM II is faster than FCM I; however, it has been demonstrated that there is a trade-off between speed and uncertainty in these methods, meaning that the FCM II presents greater uncertainties in measuring the sensor’s resistance value. A series of experiments have been carried out using an FPGA as the programmable digital device, in order to confirm the validity of both methods and evaluate the speed increase they provide, together with the errors in the results. These experiments show that depending on the choice of parameters, reductions of up to 55% can be achieved in conversion times without any appreciable increase in relative errors in the estimates of *R*.

## 8. Patents

José Antonio Hidalgo López, Jesús Alberto Botín Córdoba, Óscar Oballe Peinado, José Antonio Sánchez Durán. “Método y dispositivo para la medición de resistencias mediante un circuito de interfaz directa.” ES Patent with application number P201930781.

## Figures and Tables

**Table 1:** Measurement times for *T_Rmax_* and *T_max_* as a function of *T_x_* and of the calibration method used. The Rmax value used was 7464.5 Ω.

*T_x_*(µs)	*T_Rmax_*(µs)		*T_max_*(µs)		
	TPCM	FCM I or FCM II	TPCM	FCM I	FCM II
81.92	306.5	115.2	605.1	413.7	273.0
163.84	306.5	185.0	605.1	483.6	410.1
245.76	306.5	254.7	605.1	553.3	547.1
